# Effects of *Zataria multiflora*, *Salvia officinalis* and Probiotics on Intestinal Microbial Colonization, Immunocompetence, Jejunal Histology and Performance of Laying Hens

**DOI:** 10.1002/vms3.70147

**Published:** 2025-03-25

**Authors:** Vahid Khosravi, Amir Hossein Mahdavi, Nasim Hatamzade Esfahani

**Affiliations:** ^1^ Department of Animal Science College of Agriculture Isfahan University of Technology Isfahan Iran

**Keywords:** immunological responses, intestinal histology, laying hens, probiotic, *Salvia officinalis*, *Zataria multiflora*

## Abstract

Medicinal plants and probiotics affect the productive performance of avian species through comparable biological mechanisms, including modifications to the intestinal microbiome, alterations in histomorphology and effects on the immune system and blood metabolites. In light of this, the current study was designed and conducted to compare the efficacy of *Zataria multiflora*, *Salvia officinalis* and probiotic microorganisms on some physiological parameters and the performance of laying hens. A total of 225 white leghorn hens aged 42 weeks were randomly assigned into 9 treatments with 5 replicates of 10 birds each. Experimental treatments consisted of a control group, 0.2% and 0.4% *Z. multiflora*, 0.2% and 0.4% *S. officinalis*, 0.2% and 0.4% the combination of *Z. multiflora* and *S. officinalis* and 0.005% and 0.01% probiotic microorganisms. Our findings showed that dietary inclusion of 0.01% probiotic, 0.4% *Z. multiflora* and 0.4% *Z. multiflora + S. officinalis* treatments decreased the intestinal *Escherichia coli* (*p* < 0.01) and *Salmonella* (*p* = 0.06) enumerations and increased the lamina propria lymphoid follicle diameter (*p* < 0.01). Feeding 0.4% *Z. multiflora*, 0.4% *Z. multiflora *+ *S. officinalis* could enhance the villus height and crypt depth (*p* < 0.05). Probiotic supplementation decreased crypt depth (*p* = 0.019) and increased villus height to crypt depth ratio (*p* < 0.05). Dietary supplementation of 0.4% *Z. multiflora* and 0.01% probiotic treatments significantly showed the highest antibody titre against the Newcastle disease virus vaccine and the lowest heterophil‐to‐lymphocyte ratio (*p* < 0.05). Over the whole trial period, feeding 0.01% probiotic and 0.4% *Z. multiflora* treatments could improve the egg production percentage and feed conversion ratio (*p* < 0.05). In conclusion, the present findings indicate that the dietary administration of 0.4% *Z. multiflora* and 0.01% probiotic treatments could improve the productive performance of laying hens by strengthening both mucosal and systemic immune functions, as well as improving health indicators related to the intestinal absorptive area.

## Graphical abstract

This study aims to determine whether *Zataria multiflora*, *Salvia officinalis* and probiotics could improve gut microbiota, jejunal histology, immunological responses and performance of laying hens. Feeding 0.01% probiotics and 0.4% *Z. multiflora* decreased the intestinal *Escherichia coli* and *Salmonella* enumerations. Dietary administration of these treatments could improve the productive performance of laying hens via fortifying mucosal and systemic immune functions as well as health indices of the intestinal absorptive area.

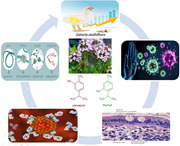

## 1 Introduction

In recent years, there has been a notable increase in research focused on alternative strategies in response to the ban and reduction of antibiotic use in the livestock industry. Natural feed additives have been identified as a viable alternative in poultry nutrition due to their effectiveness and cost efficiency (Vlaicu et al. 2023). These additives are under extensive investigation to evaluate their potential efficacy in enhancing broiler production and promoting immune function (Phillips et al. 2023). Probiotics and phytogenic additives have attracted significant attention as natural alternatives. In poultry diets, phytogenic additives serve as safe supplements that facilitate pathogen control, improve feed efficiency and increase animal production. The antioxidant properties, antimicrobial activities and anti‐inflammatory effects of these compounds are critical for enhancing overall health and performance (Galli et al. 2020). The advantageous properties of phytogenic additives are largely due to the presence of bioactive compounds, particularly phenols. Phenolic compounds are acknowledged for their diverse benefits, which include antimicrobial activity (Rafeeq et al. 2023) and considerable antioxidant capacity (Saleh et al. 2021). These compounds play a vital role in improving the health of broilers. Among them, thymol and carvacrol have been thoroughly studied for their impact on broiler well‐being. These phenolic compounds exhibit antioxidant properties that alleviate oxidative stress and promote overall health. Additionally, they possess immunomodulatory effects (Gholami‐Ahangaran et al. 2022).

*Zataria multiflora*, an aromatic medicinal species belonging to the Lamiaceae family, is distinguished by its elevated levels of thymol and carvacrol (Shomali and Mosleh 2019). This multifaceted herb is employed in both traditional medicine and culinary applications. Its use in folk medicine for a variety of health purposes has a historical background (Khazdair et al. 2022). Recent pharmacological investigations have demonstrated the notable antimicrobial properties of *Z. multiflora*, which shows efficacy against a wide range of bacterial and fungal strains (Yin et al. 2017). Furthermore, the administration of *Z. multiflora* extract to broiler chickens over a 42‐day period has been found to improve the composition of gut microbiota and indices of intestinal health (Hedayati, Khalaji, and Manafi 2022). Moreover, it has been reported that *Z. multiflora* increases feed intake and supports weight gain in broiler chickens (Talebi et al. 2021).

*Salvia officinalis*, commonly referred to as sage, is a member of the Lamiaceae family and has been widely utilized throughout history (Sharma et al. 2020). This herb is a rich source of phenolic compounds and terpenoids, making it beneficial in poultry and animal feed due to its effectiveness against bacterial infections and oxidative stress (Piesova et al. 2012). *S. officinalis* has been shown to inhibit neuronal degradation, reduce inflammatory markers and enhance immune function (Mansourabadi et al. 2016). Dietary supplementation with sage in broiler chickens has been associated with improved immune responses and a significant reduction in pathogenic bacteria, including *Escherichia coli* (Rasouli et al. 2020). Furthermore, dietary inclusion of aqueous leaf extracts of sage has demonstrated favourable effects on immune parameters and exhibited antibacterial activity in broiler chickens (Rasouli et al. 2020). *S. officinalis* has also shown inhibitory effects against various bacteria, including *Staphylococcus*, *Bacillus subtilis*, *Salmonella enteritidis*, *Candida albicans* and *Bacillus cereus* (Gharenaghadeh et al. 2017).

In light of the increasing body of research examining the use of probiotics and medicinal plants in poultry diets to enhance immune function, gastrointestinal health and productive performance, this study aimed to evaluate the effects of different levels of *Z. multiflora*, *S. officinalis*, their combination and probiotic microorganisms. The objective was to determine whether these interventions are effective and exhibit comparable efficacy in improving the immune system and gastrointestinal health status and subsequently influencing the performance characteristics of laying hens.

## 2 Materials and Methods

### 2.1 Experimental Design and Dietary Treatments

The experiment was carried out in accordance with the comprehensive animal welfare guidelines established by the Federation of Animal Science Societies (FASS, 2020). All animal care and experimental procedures were approved by the Animal Policy and Welfare Committee of the Isfahan University of Technology (IUT‐09234). Before the start of the experiment, a pretest recording period was conducted for 30 days to select laying hens with similarities in egg production levels and body weights. Assigning randomly the 225 laying hens (Hy‐Line W36 strain), 42 weeks old, into 9 treatments and having 5 replicates of 10 hens each. The basal diet was formulated using corn and soya bean meal to provide the necessary nutrient requirements of laying hens based on Hy‐Line W‐36 guidelines (Table 1). Experimental treatments were included in the control group (nonadditive on a basal diet), basal diet along with 0.2% and 0.4% *Z. multiflora*, 0.2% and 0.4% *S. officinalis*, a mixture of 0.2% and 0.4% of *Z. multiflora* and *S. officinalis*, as well as Protexin probiotic (a commercial probiotic produced by Probiotics International in England) at concentrations of 0.005% and 0.01% within the diet. It contained 2 × 10^9^ colony‐forming units (CFU)/g of beneficial bacterial species, including *Lactobacillus plantarum*, *Lactobacillus delbrueckii* subsp. *bulgaricus*, *Lactobacillus acidophilus*, *Lactobacillus rhamnosus*, *Bifidobacterium bifidum*, *Streptococcus salivarius*, *Enterococcus faecium*, *Candida pintolopesii* and *Aspergillus oryzae*. The humidity, temperature and light conditions were similar in different laying hen replicates. During the entire 10 weeks of the experimental trial, from 42 to 52 weeks of age, water and feed were supplied ad libitum.

**TABLE 1:** Ingredient and chemical composition of the basal diets.

Diet components (%)	Provided (%)	The chemical composition of the basal diet	
Corn	56.35	Metabolizable energy (kcal/kg)	2820
Soya bean meal	24.46	Crude protein (%)	15.25
Soya bean oil	3.97	Calcium (%)	4.35
Wheat bran	2.00	Available phosphorus (%)	0.46
Dicalcium phosphate	1.80	Potassium (%)	0.69
Calcium carbonate	10.21	Chlorine (%)	0.18
Common salt	0.24	Sodium (%)	0.19
dl‐Methionine	0.17	Lysine (%)	0.79
Sodium bicarbonate	0.30	Threonine (%)	0.59
Vitamin premix^a^	0.25	Methionine (%)	0.42
Mineral premix^b^	0.25	Methionine + cystine (%)	0.67

^a^ Vitamin premix provided the following per kilogram of diet: A, 10,000 IU, D3, 2500 IU; E, 10 IU; B1, 2.2 mg; B2, 4 mg; B3, 8 mg; B6, 2 mg; B9, 0.56 mg; B12, 0.015 mg; H2, 0.15 mg; Choline chloride, 200 mg.

^b^ Mineral premix provided the following per kilogram of diet: Mn, 80 mg; Fe, 50 mg; Zn, 60 mg; Cu, 5 mg; I, 1 mg and Se, 0.1 mg.

### 2.2 Antibody Response to Sheep Red Blood Cell (SRBC)

SRBCs were subjected to three washes in phosphate‐buffered saline (PBS), resulting in a final dilution of 7% (v/v) in PBS. On experimental trial days 42 and 56, two birds from each replicate were administered an intramuscular injection of 1.0 mL of a 7% suspension of SRBC. Blood samples were collected 7 and 14 days following the first and second immunizations, respectively, to assess the antibody titres in the plasma using a haemagglutination assay (Sarrami et al. 2022).

### 2.3 Production of Antibody Against Newcastle Disease (ND) Virus Vaccine

On Day 36 of the experiment, the birds were administered the ND virus vaccine via their drinking water. After 1 week from each replicate, three birds were randomly selected for wing vein bleeding. Using centrifugation for 10 min at 5000 × *g*, sera were separated and the production of antibodies against the ND virus vaccine was investigated by a haemagglutination inhibition assay (Ghasemi, Sedghi, and Mahdavi 2020).

### 2.4 Differential Counts of Leukocytes

On Day 70 of the experimental trial, randomly three birds per replicate were bled, and by screening a Gimsa‐stained slide, the differential counts of leukocytes were performed. The different leukocyte subpopulations were counted, and the ratio of heterophils to lymphocytes was calculated (Serri et al. 2022).

### 2.5 Measurement of Jejunal Morphological Alterations

At the end of the experiment, two birds per replicate were randomly selected and sacrificed to investigate intestinal histomorphology (Sarrami et al. 2022). A segment (2 cm) of the jejunal region anterior to Meckel's diverticulum was surgically removed to examine it under a light microscope. The histological sections were promptly submerged in a 100 mL/L formaldehyde solution for fixation; after the fixation process, the samples were then enclosed in paraffin wax for embedding. Thin sections, measuring 5 µm in thickness, were prepared using a microtome. These sections were then stained with haematoxylin–eosin and observed using a light microscope.

### 2.6 Intestinal Bacterial Enumeration

To count the bacterial population of the intestine, 1 g of ileal digesta content was collected from two birds of each replicate, diluted sequentially with sterile buffer PBS to a dilution of 10^−6^, and then 100 µL of each dilution with two replications was cultured on eosin methylene blue‐agar culture medium and incubated at 37°C for 24 h. Using biochemical tests of methyl red, indole, citrate reactions and Voges–Proskauer, *Salmonella enteritidis* and *E. coli* colonies were confirmed. To quantify the total number of bacterial CFU per gram of digesta, the CFU count was determined per plate using the spread plate method.

### 2.7 Performance

During the experimental trial, eggs were collected daily (at 8:00 a.m.) and weighed immediately after being collected. Egg mass was calculated utilizing both the weight and the number of eggs. The average daily feed intake was calculated using the actual daily feed intake per replicate. At 35‐day intervals, feed consumption, egg production, egg mass, egg weight and feed conversion ratio were measured.

### 2.8 Statistical Analysis

All data from each replicate were considered. The experimental units were subjected to statistical analysis using a completely randomized design and the general linear model procedure within the Statistical Analysis System (SAS 2013). The model of statistics was *Y_ij_* = *µ* + *T_i_* + *e_ij_*. *Y_ij_* is the value of each observation, *µ* is the average, *T_i_* is the effect of *i* treatment and *e_ij_* is the random error associated with each observation. A probability value of *p* < 0.05 was considered statistically significant, and *p* values between 0.05 and 0.10 were considered representative of a trend. If the differences were significant, the Tukey test compared the means.

## 3 Results

The treatment's effects on the intestinal populations of *E. coli* and *Salmonella* have been presented in Table 2. The highest populations of *Salmonella* (*p* = 0.06) and *E. coli* (*p* < 0.05) were observed in the control group and 0.005% probiotic supplementation. However, feeding 0.01% probiotic, 0.4% *Z. multiflora* and 0.4% *Z. multiflora + S. officinalis* led to a remarkable decrease in both populations.

**TABLE 2:** Effect of different levels of *Zataria multiflora*, *Salvia officinalis*, a mixture of *Z. multiflora* and *S. officinalis* and probiotics on the enumerations of laying hens ileal bacteria (Log_10_ CFU/g).

Treatment	*Escherichia coli*	*Salmonella*
Control	6.25^a^	4.75
0.2% *Zataria multiflora*	6.04^ab^	3.75
0.4% *Zataria multiflora*	5.07^c^	3.27
0.2% *Salvia officinalis*	5.25^bc^	4.07
0.4% *Salvia officinalis*	5.53^b^	4.12
0.2% *Zataria multiflora* + *Salvia officinalis*	5.75^ab^	4.10
0.4% *Zataria multiflora* + *Salvia officinalis*	5.11^c^	4.01
0.005% Probiotic	6.25^a^	4.25
0.01% Probiotic	5.07^c^	3.25
SEM	0.13	0.30
*p* value	0.03	0.06

^a–c^ Means with no common superscript within each column are significantly (*p* < 0.05) different.

As shown in Table 3, villus height, crypt depth, the ratio of villus height to crypt depth and lamina propria lymphatic follicle diameter were influenced by dietary treatments (*p* < 0.05). Dietary administration of 0.4% *Z. multiflora* and 0.4% *Z. multiflora* + *S. officinalis* showed a remarkable increase in the height of the villus (*p* = 0.019) and depth of the crypt (*p *< 0.01). Feeding 0.01% probiotic significantly decreased the depth of the crypt and consequently increased the ratio of villus height to crypt depth. Lamina propria lymphatic follicle diameter significantly increased using 0.01% probiotic, 0.4% *Z. multiflora* + *S. officinalis*, 0.4% *S. officinalis* and 0.4% *Z. multiflora* (*p* < 0.01).

**TABLE 3:** Effect of different levels of *Zataria multiflora*, *Salvia officinalis*, a mixture of *Z. multiflora* and *S. officinalis* and probiotics on the intestinal histological changes in laying hens.

Treatment	Villus height (µm)	Crypt depth (µm)	VH:CD^1^	LLF^1^ number (score)	GCN (score)	LLF diameter (µm)
Control	918.60^b^	47.48^b^	19.34^b^	^2^+3.37	+2.36	17.44^c^
0.2% *Zataria multiflora*	952.78^b^	51.69^ab^	18.43^b^	+3.50	+2.50	33.08^ab^
0.4% *Zataria multiflora*	997.67^a^	56.89^a^	17.53^b^	+3.87	+2.75	37.96^a^
0.2% *Salvia officinalis*	919.85^b^	50.48^ab^	18.22^b^	+3.25	+2.50	25.68^b^
0.4% *Salvia officinalis*	929.01^b^	50.68^ab^	18.33^b^	+3.50	+2.75	35.78^a^
0.2%*Zataria multiflora* + *Salvia officinalis*	930.47^b^	49.44^ab^	18.82^b^	+3.37	+2.37	27.93^b^
0.4% *Zataria multiflora* + *Salvia officinalis*	1017.50^a^	55.71^a^	18.26^b^	+3.75	+3.00	37.17^a^
0.005% Probiotic	917.74^b^	45.68^bc^	20.09^ab^	+3.37	+2.50	27.37^b^
0.01% Probiotic	951.95^b^	41.02^c^	23.21^a^	+3.62	+2.62	36.39^a^
SEM	14.67	2.33	1.01	0.19	0.18	0.78
*p* value	0.005	0.019	0.04	0.38	0.29	<0.001

Abbreviations: GCN, goblet cells number; LLF, laminapropria lymphatic follicles; LLF, laminapropria lymphatic follicles; VH:CD, villus height‐to‐crypt depth ratio.

^a–c^ Means with no common superscript within each column are significantly (*p* < 0.05) different.

^1^ Number of +’s indicates the severity of the histological changes.

Table 4 indicates that the blood lymphocyte count increased (*p* < 0.05), whereas the ratio of heterophils to lymphocytes significantly decreased (*p* < 0.01) following supplementation with 0.4% *Z. multiflora* and 0.01% probiotics.

**TABLE 4:** Effect of different levels of *Zataria multiflora*, *Salvia officinalis*, a mixture of *Z. multiflora* and *S. officinalis* and probiotics on differential circulating leukocyte count in laying hens (percentage).

Treatment	Heterophil	Lymphocyte	Monocyte	Eosinophil	Heterophile:Lymphocyte
Control	39.12	58.14^bc^	1.62	1.12	0.67^a^
0.2% *Zataria multiflora*	34.88	60.25^bc^	3.25	1.62	0.58^b^
0.4% *Zataria multiflora*	32.64	63.87^a^	1.62	1.87	0.51^c^
0.2% *Salvia officinalis*	35.51	61.37^ab^	2.12	1.00	0.58^b^
0.4% *Salvia officinalis*	34.50	62.00^ab^	2.12	1.37	0.56^b^
0.2% *Zataria multiflora* + *Salvia officinalis*	35.51	61.50^ab^	1.75	1.25	0.58^b^
0.4% *Zataria multiflora* + *Salvia officinalis*	34.75	61.00^ab^	3.00	1.25	0.57^b^
0.005% Probiotic	37.71	57.17^c^	3.62	1.50	0.66^a^
0.01% Probiotic	32.88	63.75^a^	2.37	1.00	0.51^c^
SEM	0.92	0.91	0.54	0.23	0.02
*p* value	0.08	0.04	0.11	0.16	0.01

^a–c^ Means with no common superscript within each column are significantly (*p* < 0.05) different.

The treatments did not have a significant impact on antibody production against SRBC during the primary immune response (Table 5). Nevertheless, dietary inclusion of 0.4% *Z. multiflora*, 0.4% *Z. multiflora* + *S. officinalis* and 0.01% probiotic resulted in a significant enhancement of antibody production in the secondary immune response to SRBC (*p* = 0.06) and the ND virus vaccine (*p* < 0.05).

**TABLE 5:** Effect of different levels of *Zataria multiflora*, *Salvia officinalis*, a mixture of *Z. multiflora* and *S. officinalis* and probiotics on the antibody titre against sheep red blood cell (SRBC) and Newcastle disease virus (Log_2_).

	Sheep red blood cells		
Treatment	Primary response	Secondary response	Newcastle disease virus
Control	5.17	4.87	6.20^b^
0.2% *Zataria multiflora*	5.87	6.15	6.62^b^
0.4% *Zataria multiflora*	6.50	6.93	8.25^a^
0.2% *Salvia officinalis*	5.87	5.61	7.07^b^
0.4% *Salvia officinalis*	6.12	6.37	7.35^ab^
0.2% *Zataria multiflora* + *Salvia officinalis*	5.87	5.12	7.11^b^
0.4% *Zataria multiflora* + *Salvia officinalis*	6.25	6.59	7.87^a^
0.005% Probiotic	6.12	5.87	6.32^b^
0.01% Probiotic	6.25	7.03	7.95^a^
SEM	0.18	0.23	0.21
*p* value	0.19	0.06	0.03

^a,b^ Means with no common superscript within each column are significantly (*p* < 0.05) different.

During the first 35 days of the experiment, no significant differences were observed among the treatments concerning performance parameters. However, the inclusion of 0.4% *Z. multiflora* and 0.01% probiotic microorganisms significantly improved egg mass, egg production and feed conversion ratio during the subsequent 35 days and throughout the entire study period (*p* < 0.05; Table 6).

**TABLE 6:** Effect of different levels of *Zataria multiflora*, *Salvia officinalis*, a mixture of *Z. multiflora* and *S. officinalis* and probiotics on the performance of laying hens.

Treatment	Daily feed intake (g)			Egg weight (g)			Egg production (%)			Egg mass (g/day per bird)			Food conversion ratio		
	0–35	35–70	0–70	0–35	35–70	0–70	0–35	35–70	0–70	0–35	35–70	0–70	0–35	35–70	0–70
Control	112.63	111.15^ab^	111.89	61.71	62.36	62.03	83.28	81.57^b^	82.42^b^	51.39	50.86	51.12^b^	2.192	2.185	2.188^a^
0.2% *Zataria multiflora*	112.01	113.73^ab^	112.87	62.20	62.76	62.48	84.14	82.41^b^	83.27^b^	52.33	51.72	52.03^b^	2.140	2.199	2.169^a^
0.4% *Zataria multiflora*	115.47	119.82^a^	117.64	64.99	65.41	65.21	87.14	86.29^a^	86.71^a^	56.63	56.44	56.53^a^	2.039	2.123	2.081^b^
0.2% *Salvia officinalis*	110.93	108.86^b^	109.89	62.73	62.04	62.38	83.42	81.00^b^	82.21^b^	52.33	50.25	51.29^b^	2.120	2.166	2.143^ab^
0.4% *Salvia officinalis*	109.14	112.74^ab^	110.94	62.06	62.57	62.31	82.71	82.52^b^	82.61^b^	51.32	51.63	51.48^b^	2.127	2.184	2.156^a^
0.2% *Zataria multiflora* *+ Salvia officinalis*	114.41	111.76^ab^	113.08	61.23	63.06	62.14	83.84	82.42^b^	83.13^b^	51.33	51.97	51.65^b^	2.229	2.150	2.190^a^
0.4% *Zataria multiflora* + *Salvia officinalis*	112.40	113.65^ab^	113.02	62.68	64.66	63.67	84.14	83.70^ab^	83.92^ab^	52.73	54.12	53.44^ab^	2.132	2.099	2.115^ab^
0.005% Probiotic	111.83	109.87^b^	110.85	62.70	63.45	63.07	84.42	82.73^ab^	83.57^ab^	52.93	52.49	52.71^b^	2.113	2.093	2.103^ab^
0.01% Probiotic	112.01	110.08^b^	111.04	62.22	63.28	62.75	85.57	84.92^a^	85.24^a^	53.24	53.74	53.49^ab^	2.104	2.048	2.076^b^
SEM	3.53	2.06	2.42	0.76	4.05	2.00	1.92	1.09	1.01	2.00	1.86	1.27	0.08	0.11	0.07
*p* value	0.33	0.02	0.05	0.56	0.74	0.66	0.26	0.03	0.04	0.08	0.07	0.04	0.06	0.83	0.03

^a–c^ Means with no common superscript within each column are significantly (*p* < 0.05) different.

## 4 Discussion

Herbal medicines, due to their extensive pharmacological and biological activity, larger safety margin and lower cost, have been used as traditional medicines to treat various health conditions (Lane and Takhar 2011). On the other hand, the known health‐improving features of probiotics, including antioxidant, antimicrobial activity, immunomodulatory properties and bioactive molecule production (Minj et al. 2021), have caused them to receive massive attention recently. In this context, the present experiment was conducted to examine the effects of two widely recognized herbs and to compare their impacts with those of probiotics on the welfare and subsequent production performance of layer hens.

*Z. multiflora* is an aromatic plant known as ‘Avishane Shirazi or Shirazi thyme’ (Ali et al. 2000; Aliakbarlu, Sadaghiani, and Mohammadi 2013). Shirazi thyme is widely recognized in traditional medicine as one of the most prominent medicinal plant varieties (Ali et al. 2000). *Z. multiflora* essential oils contain a high level of polyphenols, such as carvacrol, which exhibit antimicrobial activities by disrupting the outer membrane of gram‐negative bacteria (Chouhan, Sharma, and Guleria 2017; Heydari‐Majd et al. 2019). Thymol is another bioactive compound of *Z*. multiflora that alters the bacteria's cell wall, structural integrity and fluidity of the membrane (Di Pasqua et al. 2007). In parallel, our findings indicated that dietary supplementation of 0.4% *Z. multiflora* and 0.4% *Z. multiflora* + *S. officinalis* could suppress *Salmonella* and *E. coli* populations as gram‐negative bacteria in the intestine of laying hens. It is reported that the administration of *Z. multiflora* extract in broilers diet reduced *E. coli* and increased *Lactobacilli* populations in the ileal (Mohammadpour, Darmani‐Kuhi, and Mohiti‐Asli 2015). These findings were also observed following the administration of *Z. multiflora* extract in both high‐fat and normal diets for broilers over a 42‐day period (Nobakht, Darmani‐Kuhi, and Mohiti‐Asli 2016). The observed decrease in the intestinal pathogenic population following probiotic supplementation can be attributed to various mechanisms associated with probiotic microorganisms. These include competition for adhesion sites and nutrients, reinforcement of the intestinal epithelial barrier function, degradation and detoxification of harmful substances and immune modulatory effects.

Bioactive compounds, including thymol and carvacrol in *Z. multiflora*, have intestinal morphology‐improving activities (Bistgani et al. 2019; Seifeldeen et al. 2021). The two major indicators of digestive health and the absorption capacity of the intestine are crypt depth and villus height, whereas a higher ratio of villus height to crypt depth suggests a higher capacity for digestion and absorption, which is associated with higher feed efficiency (De Verdal et al. 2010). Carvacrol and thymol with reactive oxygen species–scavenging activity attenuate oxidative stress–induced intestinal barrier injury through PI3K/Akt‐mediated Nrf2 signalling pathway (Zhuang et al. 2019). In *Clostridium perfringens*–challenged broiler chickens, these bioactive compounds improved immune system and intestinal health via their antibacterial activity and stabilizing the gut microbiota (Du et al. 2016). An uptick in short‐chain fatty acid production coupled with a reduction in ammonia synthesis may serve as potential mechanisms through which probiotics improve intestinal histomorphology (Ramlucken et al. 2020). Furthermore, their immunomodulatory properties are associated with modulating the gene expression of the immune system in chickens (Kim et al. 2010). It is reported that a mixture of thymol and carvacrol could decrease the tumour necrotic factor (TNF)‐α gene expression and increase the interleukin (IL)‐4 gene expression in the spleen of the broilers injected with lipopolysaccharides (Du, Li, and Guo 2015). Moreover, it is demonstrated that carvacrol can suppress inflammation induced by LPS in macrophages via inhibition of ERK1/2 and NFκB pathways (Somensi et al. 2019). The observed decrease in the heterophil‐to‐lymphocyte ratio and the increase in lymphocyte count following treatment with 0.4% *Z. multiflora* and 0.01% probiotic align with findings from two studies that demonstrated that essential oils containing 5 and 707 mg/kg of carvacrol promote lymphocyte proliferation (Revajova et al. 2010; Lee et al. 2011). Heterophils are at the forefront of host immune responses against invading pathogens (Acarturk et al. 2015), and the ratio of heterophils to lymphocytes is an accepted indicator of stress. The lower ratio reflects the balance in the immune system (Zahorec 2021). In this study, using 0.4% *Z. multiflora* or 0.01% probiotic significantly increased antibody production against ND virus vaccine and SRBC, which reaffirmed their immune promotion properties.

Probiotics enhance the acquired immune response exerted by T and B lymphocytes, and immune system stimulation may be due to an increase in serum protein levels, T, and phagocytosis cells (Mazziotta et al. 2023). Probiotics improve immune response by increasing some gram‐positive bacteria, including *Bifidobacteria* and *Lactobacillus* (Hoepers et al. 2024). Additionally, herbal extracts through increasing vitamin C activity may increase antibody production (Mathivanan and Kalaiarasi 2007). Mosleh, Shomali, and Aghapour Kazemi (2013) examined the influence of *Z. multiflora* essential oil on the immune responses of chickens vaccinated against live ND. Their findings indicated an enhancement of humoral immunity accompanied by a reduction in cellular immunity. Another study ascertained the positive influence of supplementing the broiler feed with the essential oil of *Z. multiflora* on the ratio of heterophil to lymphocyte, cutaneous basophil hypersensitivity test, antibody titre against SRBC antigen and white blood cell count (Khaksar, Golian, and Kermanshahi 2012). Ghazvinian, Araghi, and Abouhosseini Tabari (2018) reported the immune‐modulating properties of *Z. multiflora* followed by its stimulating effect on the production of antibodies in response to SRBC by dietary administration of 100 mg/kg for 42 days. The immune response to *Z. multiflora* appears to be dose‐dependent. Although the administration of this herb enhances B‐cell immune responses, particularly concerning the ND virus vaccine, high doses of this herb or its phytogenic components may suppress cellular immune responses, potentially through the modulation of cytokine levels (Shomali and Mosleh 2019). Performance function promotion using 0.4% *Z. multiflora* alone or as a mixture and 0.01% probiotic probably correlated with their positive effects on the immune system, gut microbiota and gastrointestinal health indices.

Additionally, *Z. multiflora* contains phytogenic compounds and minerals and can effectively influence the performance of layer hens. Up until now, some studies have indicated the significance of probiotics in enhancing feed conversion ratios (Al‐Fatah 2020). It is important to note that for probiotics to exhibit beneficial effects, an appropriate dosage must be administered. It seems that *S. officinalis* in the mixture of 0.4% *Z. multiflora* + *S. officinalis* does not lead to incremental positive effects, and the beneficial effects of this treatment are related to the 0.4% *Z. multiflora*.

## 5 Conclusion

According to the results of this study, it is proposed that feeding 0.4% *Z. multiflora* and 0.01% probiotic through promoting the intestinal histomorphology, gut microbiota and immune system improved the well‐being of birds and consequently the productive performance of laying hens. In this regard, they can be considered an alternative to antibiotic growth‐promoter. Although these treatments are competitive in achieving the best biological responses, it is advisable to use a factorial design to identify the optimal interaction effects. To minimize logistical and practical constraints, the best synergistic effects among medicinal plants should first be established through a series of planned experiments. Subsequently, an experiment can be designed to assess the different levels of this selective treatment in combination with various levels of probiotics. Furthermore, it is recommended that future research explore the effects of the concurrent application of these substances at varying concentrations under challenging conditions.

## Author Contributions

**Vahid Khosravi**: investigation, data curation, formal analysis. **Amir Hossein Mahdavi**: funding acquisition, project administration, supervision, validation, writing–review and editing. **Nasim Hatamzade Esfahani**: writing–original draft.

## Ethics Statement

The authors confirm that the ethical policies of the journal, as noted on the journal's author guidelines page, have been adhered to and approval via the Animal Care and Use Committee of Isfahan University of Technology and Federation of Animal Science Societies under protocol (FASS, 2020) has been received. The authors confirm that they have followed EU standards for the protection of animals used for scientific purposes.

## Conflicts of Interest

The authors declare no conflicts of interest.

## Data Availability

The data that support the findings of this study are available from the corresponding author upon reasonable request.
